# Functional connectivity MRI provides an imaging correlate for chimeric antigen receptor T-cell-associated neurotoxicity

**DOI:** 10.1093/noajnl/vdad135

**Published:** 2023-10-24

**Authors:** Sophia Stoecklein, Stephan Wunderlich, Boris Papazov, Michael Winkelmann, Wolfgang G Kunz, Katharina Mueller, Katharina Ernst, Veit M Stoecklein, Viktoria Blumenberg, Philipp Karschnia, Veit L Bücklein, Kai Rejeski, Christian Schmidt, Michael von Bergwelt-Baildon, Joerg-Christian Tonn, Jens Ricke, Hesheng Liu, Jan Remi, Marion Subklewe, Louisa von Baumgarten, Florian Schoeberl

**Affiliations:** Department of Radiology, University Hospital, LMU Munich, Munich, Germany; Department of Radiology, University Hospital, LMU Munich, Munich, Germany; Department of Radiology, University Hospital, LMU Munich, Munich, Germany; Department of Radiology, University Hospital, LMU Munich, Munich, Germany; Department of Radiology, University Hospital, LMU Munich, Munich, Germany; Department of Neurology, University Hospital, LMU Munich, Munich, Germany; Department of Neurology, University Hospital, LMU Munich, Munich, Germany; Department of Neurosurgery, University Hospital, LMU Munich, Munich, Germany; German Cancer Consortium (DKTK), partner site Munich, German Cancer Research Center (DKFZ), Heidelberg, Germany; German Cancer Consortium (DKTK), partner site Munich, German Cancer Research Center (DKFZ), Heidelberg, Germany; Department of Medicine III, University Hospital, LMU Munich, Munich, Germany; Laboratory for Translational Cancer Immunology, Gene Center, LMU Munich, Munich, Germany; Department of Neurosurgery, University Hospital, LMU Munich, Munich, Germany; German Cancer Consortium (DKTK), partner site Munich, German Cancer Research Center (DKFZ), Heidelberg, Germany; Department of Medicine III, University Hospital, LMU Munich, Munich, Germany; Laboratory for Translational Cancer Immunology, Gene Center, LMU Munich, Munich, Germany; Bavarian Cancer Research Center (BZKF), Erlangen, Germany; German Cancer Consortium (DKTK), partner site Munich, German Cancer Research Center (DKFZ), Heidelberg, Germany; Laboratory for Translational Cancer Immunology, Gene Center, LMU Munich, Munich, Germany; Bavarian Cancer Research Center (BZKF), Erlangen, Germany; Department of Medicine III, University Hospital, LMU Munich, Munich, Germany; Department of Medicine III, University Hospital, LMU Munich, Munich, Germany; German Cancer Consortium (DKTK), partner site Munich, German Cancer Research Center (DKFZ), Heidelberg, Germany; Department of Medicine III, University Hospital, LMU Munich, Munich, Germany; Bavarian Cancer Research Center (BZKF), Erlangen, Germany; Department of Neurosurgery, University Hospital, LMU Munich, Munich, Germany; German Cancer Consortium (DKTK), partner site Munich, German Cancer Research Center (DKFZ), Heidelberg, Germany; Department of Radiology, University Hospital, LMU Munich, Munich, Germany; Department of Neurology, University Hospital, LMU Munich, Munich, Germany; Department of Radiology, Athinoula A. Martinos Center for Biomedical Imaging, Massachusetts General Hospital, Charlestown, MA, USA; Department of Neuroscience, Medical University of South Carolina, Charleston, SC, USA; Department of Radiology, University Hospital, LMU Munich, Munich, Germany; Department of Neurology, University Hospital, LMU Munich, Munich, Germany; German Cancer Consortium (DKTK), partner site Munich, German Cancer Research Center (DKFZ), Heidelberg, Germany; Department of Medicine III, University Hospital, LMU Munich, Munich, Germany; Laboratory for Translational Cancer Immunology, Gene Center, LMU Munich, Munich, Germany; Bavarian Cancer Research Center (BZKF), Erlangen, Germany; Department of Neurology, University Hospital, LMU Munich, Munich, Germany; Department of Neurosurgery, University Hospital, LMU Munich, Munich, Germany; German Cancer Consortium (DKTK), partner site Munich, German Cancer Research Center (DKFZ), Heidelberg, Germany; Bavarian Cancer Research Center (BZKF), Erlangen, Germany; Department of Neurology, University Hospital, LMU Munich, Munich, Germany; German Center for Vertigo and Balance Disorders, University Hospital, LMU Munich, Munich, Germany

**Keywords:** CAR T-cell therapy, EEG-coherence, immune effector cell-associated neurotoxicity syndrome, resting-state fMRI, whole-brain connectivity and dysconnectivity

## Abstract

**Background:**

Treatment of hematological malignancies with chimeric antigen receptor modified T cells (CART) is highly efficient, but often limited by an immune effector cell-associated neurotoxicity syndrome (ICANS). As conventional MRI is often unremarkable during ICANS, we aimed to examine whether resting-state functional MRI (rsfMRI) is suitable to depict and quantify brain network alterations underlying ICANS in the individual patient.

**Methods:**

The dysconnectivity index (DCI) based on rsfMRI was longitudinally assessed in systemic lymphoma patients and 1 melanoma patient during ICANS and before or after clinical resolution of ICANS.

**Results:**

Seven lymphoma patients and 1 melanoma patient (19–77 years; 2 female) were included. DCI was significantly increased during ICANS with normalization after recovery (*P* = .0039). Higher ICANS grades were significantly correlated with increased DCI scores (*r* = 0.7807; *P* = .0222). DCI increase was most prominent in the inferior frontal gyrus and the frontal operculum (ie, Broca’s area) and in the posterior parts of the superior temporal gyrus and the temporoparietal junction (ie, Wernicke’s area) of the language-dominant hemisphere, thus reflecting the major clinical symptoms of nonfluent dysphasia and dyspraxia.

**Conclusions:**

RsfMRI-based DCI might be suitable to directly quantify the severity of ICANS in individual patients undergoing CAR T-transfusion. Besides ICANS, DCI seems a promising diagnostic tool to quantify functional brain network alterations during encephalopathies of different etiologies, in general.

**Key Points**

Resting-state functional MRI (RsfMRI)-based dysconnectivity index (DCI) retraced clinical immune effector cell-associated neurotoxicity syndrome (ICANS) course in each individual patient (ie, it was elevated in ICANS and decreased when symptoms resolved).
DCI was significantly associated with ICANS grading.
DCI depicted the most profound connectivity disruptions in cerebral hubs for language processing and multistage actions, resembling the core symptoms of ICANS.

**Importance of the Study**

Immunotherapy with chimeric antigen receptor (CAR) T cells is one of the most innovative and promising treatments for cancer. Despite its success, its application is limited by neurotoxic side effects. There is an unmet clinical need for an objective, quantifiable, and readily available marker to assess immune effector cell-associated neurotoxicity syndrome (ICANS) in the individual patient, in order to inform underlying disease mechanisms, detect individuals at risk for developing ICANS, monitor patients with ICANS, and predict their course of recovery. As conventional structural brain MRI is mostly unremarkable in ICANS, we applied functional MRI and showed that resting-state functional MRI (rsfMRI)-based dysconnectivity index (DCI) can capture and quantify ICANS-related connectivity alterations in the individual patient. The results of our piloting case series propose that rsfMRI-based DCI is a promising imaging marker that should be systematically explored in prospective studies of patients undergoing CART therapy.

Chimeric antigen receptor T cells (CAR T) represent a powerful new class of adoptive immunotherapy for the treatment of relapsed or refractory hematological malignancies and also solid cancer entities.^1^ However, its success is hampered by immune-mediated adverse events, including immune effector cell-associated neurotoxicity syndrome (ICANS). ICANS affects up to two-thirds of such patients, clinically often manifesting with nonfluent dysphasia and dyspraxia for multistage actions, without structural lesions on conventional MRI.^2,3^ The exact pathophysiology of ICANS remains puzzling and, among other aspects, the extent to which ICANS is associated with disruptions of dedicated neuronal networks is yet to be determined.

Based on resting-state functional MRI (rsfMRI), we recently developed an imaging tool, called the dysconnectivity index (DCI), which allows for the reliable quantification of functional connectivity disruptions in individual patients by comparing the connectivity profile of each voxel of an individual patient to a reference profile of healthy controls.^4^ In a recent study in glioma patients this approach revealed network alterations in distant, non-lesional brain tissue. The DCI, as a quantitative measure of these alterations in each individual patient, was correlated with underlying tumor biology and cognitive performance.^4^

In this proof-of-concept study, we utilized rsfMRI to analyze whether the DCI is increased in ICANS and associated with ICANS grade. We furthermore aimed to depict cortical brain regions that might be predominantly affected by ICANS-associated connectivity alterations.

## Material and Methods

The sample consisted of 8 patients (19–77 years; female: 2; 7 right handed), among whom 7 had systemic lymphomas, and 1 had metastasized melanoma. All of these patients developed ICANS after the administration of CAR T cells (see Table 1). None of the patients had antecedent or present disease involvement of the nervous system. For further clinical characterization of the sample, please refer to Table S1. ICANS was graded according to current guidelines (ASTCT).^2^ 3 Tesla structural and rsfMRI according to the protocol of the Brain Genomics Superstruct Project (GSP)5 was performed in each patient at 2 different time points: at baseline before CAR T-transfusion (pre-ICANS, *n* = 3) or after clinical recovery from ICANS (post-ICANS, *n* = 5) and during maximum ICANS (ICANS, *n* = 8). The control cohort consisted of 50 healthy subjects (range: 27–35 years: female: 56.0%), collected as part of the GSP.^5^ For each subject, a correlation matrix of 32,492 cortical voxels in each hemisphere was calculated and normalized using *z*-score.

**Table 1.** Patient characteristics

Patient	#1	#2	#3	#4	#5	#6	#7	#8
Age (years)	77	70	72	19	36	70	66	70
Sex	m	m	f	m	m	m	m	f
Handedness	right	right	both	right	right	right	right	right
Diagnosis	MCL Stadium IVB	DLBCL	MCL Stadium IVA	DLBCL	DLBCL	DLBCL	Malignant melanoma Stage IV	DLBCL Stage IV
CART product	KTE-X19	Tisa-cel	KTE-X19	Axi-cel	Tisa-cel	Tisa-cel	MB-CART	Axi-cel
CRS grade (ASTCT)	2	2	2	2	3	3	2	1
CRS duration (days)	13	6	10	5	6	14	5	5
ICANS grade (ASTCT)	4	2	1	3	1	2	4	3
ICE score and symptoms	0^a,b,c,d^	6^a,b,c,e^	8^a,b,c^	2^a,b,c,d,e,f^	8^a,b,c^	6^a,b,c,e^	0^a,b,c,d^	7^a,b,c^
ICANS duration (days)	11	7	9	15	3	8	9	6

^a^Dysphasia (object naming; spontaneous speech); ^b^ dyspraxia for multistage actions (making a phone call; writing a handy message); ^c^ dyscalculia (100–7, etc.); ^d^ decreased vigilance state (ie, sopor, coma); ^e^ postural and action tremor; ^f^ epileptic seizure.

Abbreviations: Axi-cel, axicabtagene ciloleucel; DLBCL, diffuse large B-cell lymphoma; MCL, mantle cell lymphoma; Tisa-cel, tisagenlecleucel; pre-ICANS (before CAR T-transfusion); maximum ICANS (during maximum neurotoxicity after CAR T-transfusion); post-ICANS (full recovery from ICANS after CAR T-transfusion); n.a., not available

The DCI was calculated as previously described by Stoecklein et al.^4,6^ Briefly, connections that diverge beyond a certain threshold (for this study 16 standard deviations) from the distribution of the respective connection in the reference group, are counted as “dysconnected.” The individual patient’s dysconnectivity count (DCC) is then summarized in each hemisphere, and normalized to the number of voxels in the respective hemisphere, resulting in the DCI. To evaluate the difference of DCI during versus before or after ICANS, the Wilcoxon matched-pairs signed-rank test was used. Correlation between ICANS grade and DCI was calculated using Spearman rank correlation. A significance level of *P* < .05 was applied. Statistical analysis was performed using SAS version 9.4, Copyright SAS Institute Inc., Cary, NC.

The study was approved by the local ethics committee of the Ludwig Maximilians University (No: 20-646; 19-817) and conducted in accordance with the Declaration of Helsinki. Written informed consent was obtained from each participant.

## Results

**Figure 1.**
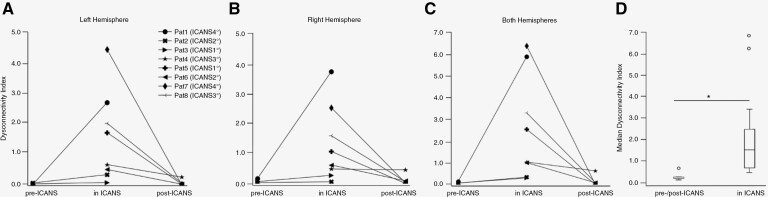
Intraindividual changes of the dysconnectivity indices during immune effector cell-associated neurotoxicity syndrome (ICANS). The x-axes show the scanning timepoints, the y-axes display the extent of dysconnectivity in each patient. (A) The dysconnectivity index (DCI) of the left hemisphere during ICANS was increased in all patients, when compared to either the baseline DCI (ie, before CAR T-transfusion, *n* = 3) or the follow-up after clinical recovery from ICANS (*n* = 5). Clinical severity of ICANS (ie, ICANS grade) as measured by the ICE score showed overall good correspondence with DCI values during maximum ICANS except for patient (patient #5), who exhibited a marked increase of DCI despite mild clinical neurotoxic symptoms (ie, ICANS °1). (B) The DCI of the right hemisphere was increased in all patients during ICANS (*n* = 8). After clinical recovery from ICANS, DCI was normalized with 1 exception (ie, patient #4) exhibiting continued elevation of DCI. (C) The whole-brain DCI during maximum ICANS was defined as the sum of DCIs of the left and right hemispheres. (D) Statistical analysis revealed a significant increase of DCI during ICANS as compared to DCI at baseline before CAR T-transfusion (*n* = 3) or after clinical recovery from ICANS (*n* = 5). Asterisk indicates *P* < .05.

In all 8 patients, whole-brain DCI was increased during ICANS as compared to the pre- or post-ICANS scan with an overall significantly elevated DCI during ICANS (*P* = .0039; see Figure 1 and Table 2). Higher ICANS grades were significantly correlated with a higher DCI on a whole brain level (*r* = 0.7807, *P* = .0222), and with the DCI of the left hemisphere (*r* = 0.7807, *P* = .0222), but not with the DCI of the right hemisphere (*r* = 0.6831, *P* = .0618).

**Table 2.** Cortical findings

Patient	#1		#2		#3		#4		#5		#6		#7		#8	
DCI: rsfMRI data																
Hemisphere	L	R	L	R	L	R	L	R	L	R	L	R	L	R	L	R
Pre-ICANS	0.04	0.17	0.09	0.04	0.06	0.08	n.a.	n.a.	n.a.	n.a.	n.a.	n.a.	n.a.	n.a.	n.a.	n.a.
in ICANS	2.61	3.55	0.35	0.07	0.10	0.28	0.66	0.47	1.67	1.03	0.51	0.60	4.28	2.41	1.96	1.53
Post-ICANS	n.a.	n.a.	n.a.	n.a.	n.a.	n.a.	0.27	0.45	0.05	0.02	0.05	0.10	0.04	0.04	0.09	0.08
Absolute change	+2.57	+3.38	+0.26	+0.03	+0.04	+0.20	-0.39	-0.02	-1.62	-1.01	-0.46	-0.50	-4.24	-2.37	-1.86	-1.45
Change in %	+6425	+1988	+289	+75	+67	+250	-144	-4	-3240	-5050	-902	-500	-10600	-5925	-2077	-1713

EEG data																
Pre-ICANS	11 Hz, normal		9 Hz, normal		11 Hz, normal		8 Hz, normal		10 Hz, normal		10 Hz, normal		9 Hz, normal		9 Hz, normal	
Maximum ICANS	7 Hz, continuous slowing, generalized, encephalopathic, changes °II		7 Hz, continuous, slowing, generalized, triphasic waves, encephalopathic changes °II-III		8 Hz, normal		7 Hz, intermittent slowing, generalized, encephalopathic changes °I		6-7 Hz, continuous and intermittent, slowing, generalized, encephalopathic changes °II		7 Hz, continuous slowing, generalized, encephalopathic changes °I		7 Hz, intermittent, slowing right-sided frontal, encephalopathic changes °I		8 Hz, intermittent, slowing left-sided temporal	
Post-ICANS	n.a.		n.a.		n.a.		n.a.		10 Hz, normal		n.a.		5 Hz, encephalopathic changes °II		n.a.	

Abbreviations: Pre-ICANS (before CAR T-transfusion); maximum ICANS (during maximum neurotoxicity after CAR T-transfusion); post-ICANS (full recovery from ICANS after CAR T-transfusion); n.a., not available.

Remarkably, patient #5 displayed quite a high DCI score despite only mild neurotoxicity (ie, °I, see Table 2), and patient #4 showed no complete normalization of connectivity during follow-up (see Table 2 and Figure 1).

Seven of 7 patients were right handed. In all 7 right-handed patients, relative DCI increase during ICANS was most pronounced in the left hemisphere (see Figure 1 and Table 2). Patient #3, being left handed by birth, showed the most pronounced DCI increase during ICANS in the right hemisphere.

**Figure 2.**
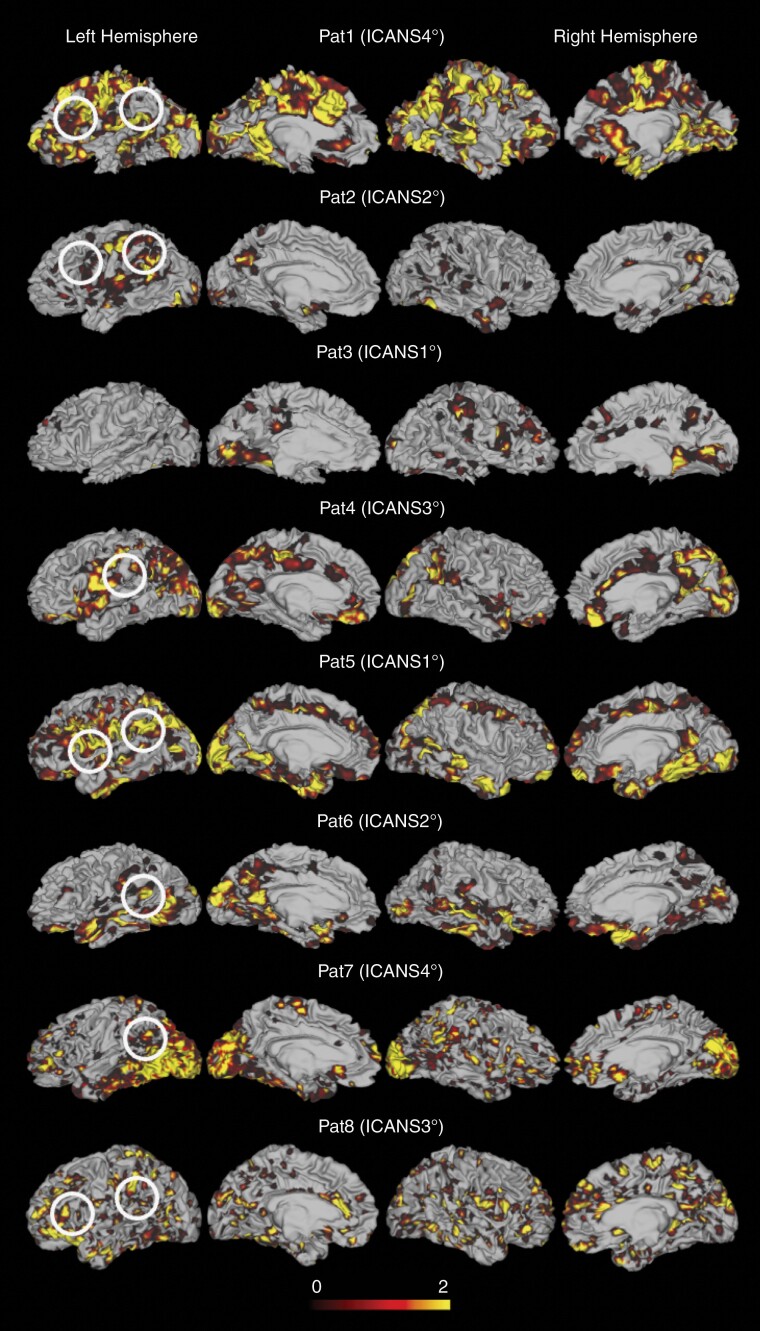
Dysconnectivity maps displaying brain regions of altered connectivity during immune effector cell-associated neurotoxicity syndrome (ICANS) for each patient. For each cortical voxel, the normalized dysconnectivity count (DCC) is displayed. Lighter color indicates a higher count of disrupted connections of the respective voxel, darker color indicates a lower count of disrupted connections. The white circles indicate cerebral hubs engaged in language processing. Disruptions in connectivity in these regions may contribute to the main symptoms of ICANS. Patients with more severe ICANS display widespread alterations of functional connectivity (eg, see patient #1 and patient 7 with ICANS °IV), while patients with mild symptoms display less connectivity changes (eg, see patient #3). As an exception, patient #5 showed widespread connectivity changes despite only mild ICANS (ie, ICANS °I). The core regions affected in each patient include the inferior frontal gyrus and frontal operculum (ie, Broca´s area, see white circles), the posterior parts of the superior temporal gyrus and temporoparietal junction (ie, Wernicke’s area, see white circles) and the supramarginal gyrus. All right-handed patients show marked changes in the dominant left hemisphere. Subject #3, who is left handed/ambidextrous shows more pronounced alterations in the right hemisphere.

The most pronounced connectivity increase during ICANS was visualized in the inferior frontal gyrus and the frontal operculum (ie, Broca’s area), the posterior parts of the superior temporal gyrus, the temporoparietal junction (ie, Wernicke’s area), and the supramarginal gyrus in the inferior parietal lobe (see Figures 2A, B).

## Discussion

In the absence of structural abnormalities on conventional MRI, rsfMRI-based DCI was increased during ICANS, and the extent of connectivity changes was significantly correlated with the severity of ICANS as assessed by ICANS grading. Interestingly, DCI increase during ICANS was particularly pronounced in the language-dominant hemisphere in all 8 patients. The prominent connectivity alterations in critical hubs of language processing and multistage actions (ie, Broca´s and Wernicke’s area, supramarginal gyrus, and temporoparietal junction) in the language-dominant hemisphere plausibly reflect the clinical core symptoms of nonfluent dysphasia and dyspraxia for multistage actions.^7^ Furthermore, these findings are in line with marked hypometabolism particularly in frontal cortical regions reported in single cases where [18F]FDG-PET/CT was performed during ICANS.^8^

In 1patient (#5), however, we found pronounced DCI despite only mild ICANS (°I), which might be due to ICANS grading by the Immune Effector Cell-associated Encephalopathy (ICE) Score, a bedside screening tool similar to the MMSE, with obvious limitations in detecting subtle cognitive impairments, particularly in younger subjects. In 1 patient (#4), the elevated DCI persisted during follow-up. This patient developed metabolic encephalopathy with seizures due to progressive systemic lymphoma. Furthermore, a relapse of ICANS cannot be definitively ruled out in this patient.

Apart from the limited sample size, the lack of post-ICANS neuropsychological testing to assess recovery constitutes a limitation of this pilot study. Future prospective studies including non-ICANS and CRS comparators as well as detailed and repeated neuropsychological assessment are warranted to further establish the clinical potential of this imaging tool.

Collectively, rsfMRI-based DCI might be suitable to directly quantify the severity of ICANS in individual patients undergoing CAR T-transfusion. Besides ICANS, DCI seems a promising diagnostic tool to quantify functional brain network alterations during encephalopathies of different etiologies, in general.

## Supplementary Material

vdad135_suppl_Supplementary_Table_S1
