# Supplementary material for: Functional connectivity MRI provides an imaging correlate for chimeric antigen receptor T-cell-associated neurotoxicity
Source: Neurooncol Adv. 2023 Oct 24;5(1):vdad135. doi: 10.1093/noajnl/vdad135 (PMC10673700; doi:10.1093/noajnl/vdad135)
Supplement: vdad135_suppl_Supplementary_Table_S1 [file vdad135_suppl_supplementary_table_s1.docx]

**Supplemental Table 1.** Clinical patient and disease characteristics

| **Patient** | **#1** | **#2** | **#3** | **#4** | **#5** | **#6** | **#7** | **#8** |
| --- | --- | --- | --- | --- | --- | --- | --- | --- |
| **Age (years)** | 77 | 70 | 72 | 19 | 36 | 70 | 66 | 70 |
| **Sex** | m | m | f | m | m | m | m | f |
| **Diagnosis** | MCL  Stage IVB | DLBCL | MCL  Stage IVA | DLBCL | DLBCL | DLBCL | Malignant melanoma  Stage IV | DLBCL  Stage IV |
| **CART product** | KTE-X19 | Tisa-cel | KTE-X19 | Axi-cel | Tisa-cel | Tisa-cel | MB-CART | Axi-cel |
| **CRS grade (ASTCT)** | 2 | 2 | 2 | 2 | 3 | 3 | 2 | 1 |
| **CRS duration (days)** | 13 | 6 | 10 | 5 | 6 | 14 | 5 | 5 |
| **ICANS grade (ASTCT)** | 4 | 2 | 1 | 3 | 1 | 2 | 4 | 3 |
| **ICANS duration (days)** | 11 | 7 | 9 | 15 | 3 | 8 | 9 | 6 |
| **Prior therapies** | | | | | | | | |
| **≥3 prior lines of therapy** | yes | no | no | yes | yes | yes | yes | no |
| **Refractory to last line of therapy before apheresis** | yes | yes | no | yes | yes | yes | yes | yes |
| **Prior autologous SCT** | no | no | no | no | yes | no | no | no |
| **Prior allogeneic SCT** | no | no | no | no | no | no | no | no |
| **Disease status at CART therapy** | SD | PD | PD | PD | PD | SD | PD | SD |
| **Baseline laboratory findings before CART therapy** | | | | | | | | |
| **LDH [<249U/l]** | 171 | 493 | 169 | 918 | 177 | 161 | 1565 | 256 |
| **CRP [<0,5 mg/dl]** | 0,4 | 3,8 | 0,2 | 10,7 | 0,2 | 0,2 | 1,5 | 3,2 |
| **Ferritin [<250ng/ml]** | 462 | 3554 | 17 | 576 | 1323 | 469 | 252 | 570 |
| **ECOG at CART therapy** | 1 | 0 | 0 | 1 | 0 | 0 | 0 | 0 |
| **IPI at CART therapy** | 1 | 4 | 2 | 4 | 2 | 1 | 1 | 2 |
| **Peak laboratory findings during ICANS** | | | | | | | | |
| **CRP [<0,5 mg/dl]** | 8,1 | 10,7 | 8 | 13,8 | 18,9 | 18,9 | 16,6 | 5,9 |
| **Ferritin [<250ng/ml]** | 2807 | 52074 | 130 | 867 | >8000 | 2228 | 1689 | 743 |
| **Il-6 [<5,9pg/ml]** | 17590 | 25227 | 2903 | 2703 | 28185 | 2036 | 3586 | 776 |
| **Management of CRS** | a | a,b | a | a,b | a,b | a,b | a,b | a |
| **Management of ICANS** | b,c | b,c | e | b | e | b | b,c,d | b |
| **Best response status post-CART therapy** | PR | PD | CR | SD | CR | SD | PR | PR |
| **PFS [days]** | 561 | 22 | 369 | 71 | 343 | 105 | 79 | 86 |

*Abbreviations:* Axi-cel: axicabtagene ciloleucel; DLBCL: diffuse large B cell lymphoma; MCL: mantle cell lymphoma; Tisa-cel: tisagenlecleucel; SCT, stem cell transplant; CR, complete remission; PR, partial remission; SD, stable disease; PD, progressive disease; LDH, lactate dehydrogenase; CRP, c-reactive protein, ECOG, Eastern Cooperative Oncology Group score; IPI, International Prognostic Index score; ^a^Tocilizumab, ^b^Dexamethasone; ^c^Methylprednisolone, ^d^Anakinra; ^e^clinical observation only; PFS, progression free survival.
